# Remote pulmonary artery pressure‐guided management of patients with heart failure: A clinical consensus statement of the Heart Failure Association (HFA) of the ESC

**DOI:** 10.1002/ejhf.3619

**Published:** 2025-04-27

**Authors:** Antoni Bayes‐Genis, Matteo Pagnesi, Pau Codina, William T. Abraham, Offer Amir, Rudolf A. de Boer, Jasper J. Brugts, Ovidiu Chioncel, Finn Gustafsson, JoAnn Lindenfeld, Wilfried Mullens, Mark C. Petrie, Giuseppe Rosano, Marco Metra

**Affiliations:** ^1^ Heart Institute, Hospital Universitari Germans Trias i Pujol, Badalona, CIBERCV Universitat Autònoma de Barcelona Barcelona Spain; ^2^ Department of Medical and Surgical Specialties, Radiological Sciences, and Public Health, Institute of Cardiology, ASST Spedali Civili University of Brescia Brescia Italy; ^3^ Division of Cardiovascular Medicine and the Davis Heart and Lung Research Institute The Ohio State University College of Medicine Columbus OH USA; ^4^ Hadassah Medical Center, Faculty of Medicine Hebrew University Jerusalem Israel; ^5^ Erasmus MC, Cardiovascular Institute Thorax Center, Department of Cardiology Rotterdam The Netherlands; ^6^ Emergency Institute for Cardiovascular Diseases ‘Prof. C.C. Iliescu’ University of Medicine Carol Davila Bucharest Romania; ^7^ Department of Cardiology, Rigshospitalet University of Copenhagen Copenhagen Denmark; ^8^ Vanderbilt Heart and Vascular Institute Vanderbilt University Medical Center Nashville TN USA; ^9^ Ziekenhuis Oost‐Limburg, Department of Cardiology Genk Belgium; ^10^ School of Cardiovascular and Metabolic Health University of Glasgow Glasgow UK; ^11^ St George's University Medical School of London London UK

**Keywords:** Heart failure, Haemodynamic monitoring, Remote monitoring, Pulmonary artery pressure, Heart Failure Association, Consensus statement

## Abstract

Episodes of worsening heart failure (HF) are a major cause of unplanned hospitalizations. Their onset is usually preceded by an early increase in intracardiac pressures with subsequent worsening of symptoms due to congestion. Implantable devices allowing daily remote pulmonary artery pressure (PAP) monitoring are useful to identify early haemodynamic changes so that medical therapy can be adjusted at an early stage, before symptom onset, and HF‐related hospitalizations be prevented. Second, the use of these devices may help to maintain clinical stability keeping PAP in the target range on a day‐to‐day basis. The CardioMEMS system allows remote PAP monitoring, and PAP‐guided medical therapy has reduced HF‐related hospitalizations in prospective, randomized, controlled clinical trials in symptomatic patients with HF, independent of their left ventricular ejection fraction. The safety and feasibility of other devices, like the Cordella implantable PAP sensor, have also been demonstrated and clinical usefulness in larger patient populations is currently being assessed in several trials. Most of the studies testing remote PAP monitoring were reported after the 2021 European Society of Cardiology HF guidelines. An update of the clinical significance and potential implications for clinical practice of these systems seems therefore warranted. The aim of this clinical consensus statement is to summarize current knowledge on remote PAP‐guided management of patients with HF, with a special focus on current evidence from clinical trials, potential impact on clinical practice and management aspects.

## Introduction

Although the treatment of heart failure (HF) has dramatically improved in the last decades, patients with HF are still burdened by a high risk of death and of episodes of worsening HF, frequently leading to hospitalizations.^1^, ^2^ Irrespective of left ventricular ejection fraction (LVEF), an increase in intracardiac pressures has a crucial role in the pathophysiology of worsening HF, driving congestion and eventually leading to overt HF decompensation.^3^ An increase in pulmonary artery pressure (PAP), that is, ‘haemodynamic’ congestion, usually precedes overt signs and symptoms of ‘clinical’ congestion by several days to weeks.^4^, ^5^, ^6^ Congestion may also cause multi‐organ failure including acute kidney injury, liver congestion and increased gut permeability and dysbiosis. Congestion may by itself, via increasing end‐diastolic volume of the ventricles, further aggravate biventricular dysfunction and decrease lymphatic drainage to the systemic venous circulation. All of the above may have a central role in further clinical decompensation and is accompanied by diuretic resistance, inflammatory and neurohormonal activation.^7^, ^8^, ^9^, ^10^ Along with optimization of guideline‐directed medical therapy (GDMT) and regular follow‐up, early detection and treatment of subclinical or overt congestion may, thus, prevent unplanned HF‐related visits and hospitalizations and possibly delay development of end‐stage HF.^3^, ^11^

Traditional follow‐up strategies in patients with HF include regular home weight control, outpatient follow‐up visits, ultrasound imaging to assess congestion (including both echocardiography, lung ultrasonography, and non‐cardiac multi‐organ assessment) and measurements of plasma concentrations of biomarkers (namely, natriuretic peptides in clinical practice).^12^, ^13^, ^14^, ^15^, ^16^ Telemedicine is of further help.^14^, ^17^, ^18^ However, biomarker measurements and ultrasound examinations can be repeated only periodically and clinical signs, including body weight, have poor sensitivity to detect congestion at an early stage.^1^, ^19^ Thus, early detection of PAP increase allows the identification of haemodynamic congestion several days to weeks before the clinical onset of the overt decompensation event. This strategy should lead to prompt adjustment of medical therapy, thus preventing worsening HF events and HF‐related hospitalizations.^3^ Implantable systems can combine continuous PAP measurement with daily transmission of recordings to healthcare providers, allowing remote PAP‐guided haemodynamic telemonitoring in patients with HF. Additionally, a more comprehensive approach is feasible when other vital signs such as daily weights, oxygen saturation and blood pressure are included in the remote monitoring.

The usefulness of invasive haemodynamic monitoring was first demonstrated in the CardioMEMS Heart Sensor Allows Monitoring of Pressure to Improve Outcomes in New York Heart Association (NYHA) Class III Heart Failure Patients (CHAMPION) trial, and these data were included in the 2021 European Society of Cardiology (ESC) guidelines on HF.^1^ However, other trials were completed after the 2021 ESC HF guidelines and could not be included. The aim of the present clinical consensus statement of the Heart Failure Association (HFA) of the ESC is therefore to discuss remote PAP‐guided management of patients with HF, ranging from available published evidence to real‐world application and management of this technology in daily clinical practice, with a major focus on currently available devices.

## Evidence from randomized controlled clinical trials

The first randomized controlled clinical trial evaluating implantable haemodynamic monitoring in HF was the Chronicle Offers Management to Patients with Advanced Signs and Symptoms of Heart Failure (COMPASS‐HF) study that tested the Chronicle system (Medtronic, Minneapolis, MN, USA), a device that measures right ventricular pressures.^20^ COMPASS‐HF showed that it was feasible and safe to remotely assess right ventricular systolic and diastolic pressures, heart rate, and pressure derivatives, but did not demonstrate a significant benefit of this monitor‐guided care versus usual care in reducing HF‐related events.^20^ Several reasons may have determined the neutral results of the COMPASS‐HF trial: the potential efficacy of pressure‐guided management was greatest in NYHA class III patients, whereas NYHA class IV patients that were included did not benefit from this strategy; the treatment algorithm in the experimental arm was not detailed and established enough, without pre‐specified pressure targets; pressure‐guided management was effective only if the investigators actually modified medical therapy in response to PAP; diuretic resistance and poor renal function may have impaired the effectiveness of outpatient optimization of diuretic therapy in response to PAP.^20^, ^21^

CardioMEMS (Abbott Laboratories, Abbott Park, IL, USA) is a PAP sensor that allows for daily remote PAP monitoring and is now available for clinical practice as approved by major regulatory bodies in Europe and the United States. Three randomized controlled trials tested its efficacy in patients with HF (*Table* *1*). The CHAMPION trial enrolled 550 patients with chronic HF, NYHA class III and a HF hospitalization in the previous 12 months, irrespective of LVEF.^22^, ^23^ After device implantation, patients were randomized to PAP‐guided management (treatment arm) or standard care with sensor data unavailable to clinicians (control arm). Device implantation was safe with close to zero pressure‐sensor failure. The primary endpoint of total HF hospitalizations at 6 months was significantly reduced in the treatment versus control arm, as well as secondary endpoints including mean PAP (mPAP) change and quality of life (QoL) at 6 months.^22^ Frequent adjustments in medical therapy, including diuretics, vasodilators and neurohormonal antagonists, led to the clinical benefits observed with CardioMEMS. Changes in diuretic administration and doses played a major role with, interestingly, both an increase and a decrease in diuretic doses occurring more often in patients in the invasive monitoring arm.^24^ At the conclusion of the randomized access period, PAP information became available to guide medical therapy also in the former control group (open access period), resulting in a significant reduction in HF hospitalizations as compared to the previous randomized phase among these patients.^23^

**Table 1:** Major clinical studies on the pulmonary artery pressure monitoring CardioMEMS system

Study	Design	Enrolment period (years)	Patients, *n*	Treatment arm	Control arm	NYHA class	≥1 HFH in the last year	LVEF ≤40%	Primary endpoint results	Other key findings
CHAMPION^22^, ^23^	Multicentre RCT (single‐blind)	2007–2009	550	Sensor implant + monitoring	Sensor implant, no monitoring	100% NYHA III	100%	78%^a^	Significant reduction in total HFH at 6 months	Significant reduction in total HFH during the entire follow‐up (15 ± 7 months) Greater reduction in mPAP and better QoL (MLHFQ) at 6 months 98.6% freedom from DSRC, 100% freedom from sensor failure After the randomized access period, reduction in total HFH in the former control group during the open access period (2010–2012, PAP information available)
GUIDE‐HF^25^	Multicentre RCT (single‐blind)	2018–2019	1000	Sensor implant + monitoring	Sensor implant, no monitoring	30% NYHA II 65% NYHA III 5% NYHA IV	55%	53%	Non‐significant difference in all‐cause death or total HFE at 12 months	Non‐significant difference in all‐cause death, total HFE and total HFH at 12 months (individual endpoints) Significant reduction in all‐cause death or total HFE at 12 months in the pre‐COVID‐19 sensitivity analysis, driven by a lower risk of total HFE and total HFH Greater reduction in mPAP at 12 months, but no significant differences in QoL endpoints (KCCQ‐12, EQ‐5D‐5L, 6MWT) at 12 months 99% freedom from DSRC
MONITOR‐HF^28^	Multicentre RCT (open‐label)	2019–2022	348	Sensor implant + monitoring	No implant	100% NYHA III	100%^b^	72%	Significantly greater change in KCCQ‐OSS from baseline to 12 months	Lower likelihood of ≥5‐point and ≥10‐point deterioration and higher likelihood of ≥5‐point and ≥10‐point improvement in KCCQ‐OSS at 12 months Significant reduction in total HFH or urgent visits during the entire follow‐up (1.8 ± 0.9 years) Greater increase in KCCQ‐CSS, KCCQ‐TSS, EQ‐5D‐5L VAS and 6MWT scores from baseline to 12 months Significant reduction in mPAP and NT‐proBNP at 12 months (in the treatment arm) 97.7% freedom from DSRC, 98.8% freedom from sensor failure
MEMS‐HF^36^, ^37^	Prospective, non‐randomized, single‐arm, multicentre study	2016–2018	239	Sensor implant + monitoring	N/A	100% NYHA III	100%	73%^a^	98.3% freedom from DSRC and 99.6% freedom from sensor failure at 12 months (co‐primary safety endpoints)	Significant reduction in annualized HFH rate in the 12 months post‐ versus pre‐implant All‐cause mortality 13.8% at 12 months (no deaths related to device, delivery system, or protocol‐required procedure) Significant reduction in dPAP, sPAP and mPAP from baseline to 12 months Mean patient adherence to daily PAP transmissions 78.1 ± 23.5%, weekly compliance 89.7 ± 17.8%, caregiver adherence to weekly review of PAP data 89.8 ± 18.7% Significant improvement in QoL endpoints (KCCQ‐OSS, KCCQ‐CSS, EQ‐5D‐5L VAS, PHQ‐9 sum score) from baseline to 12 months Clinical benefits irrespective of age, sex, LVEF, aetiology, comorbidities, and presence or subtype of PH (IpcPH, CpcPH)
CardioMEMS Post‐Approval Study^38^, ^39^, ^40^, ^50^	Prospective, non‐randomized, single‐arm, multicentre study	2014–2017	1200	Sensor implant + monitoring	N/A	100% NYHA III	100%	53%^a^	Significant reduction in annualized HFH rate in the 12 months post‐ versus pre‐implant	99.6% freedom from DSRC and 99.9% freedom from sensor failure at 2 years (primary safety endpoints) Reduction in HFH rate sustained at 2 years post‐implant All‐cause mortality 16% at 12 months and 29% at 2 years Significant reduction in dPAP and mPAP from baseline to 12 months and 2 years Mean daily PAP transmissions 76 ± 24%, mean weekly PAP transmissions 93 ± 16% Clinical benefits irrespective of sex, race, LVEF, aetiology, obesity and renal function
COAST‐UK^41^	Prospective, non‐randomized, single‐arm, multicentre study	2017–2018	100	Sensor implant + monitoring	N/A	100% NYHA III	100%	N/A	100% freedom from DSRC and 99% freedom from sensor failure at 2 years (primary safety endpoints)	Significant reduction in annualized HFH rate in the 12 months post‐ versus pre‐implant All‐cause mortality 10% at 12 months Significant reduction in dPAP, sPAP and mPAP from baseline to 12 months Mean daily home upload compliance rate 85.9 ± 19.3%, weekly upload compliance 94.5 ± 14.2% Non‐significant change in EQ‐5D‐5L VAS from baseline to 12 months NYHA class improvements at 12 months
Heywood *et al*.^42^	Retrospective study (Merlin.net database)	2014–2016	2000	Sensor implant + monitoring	N/A	N/A	N/A	66%^a^	Sustained mPAP reduction up to 6 months	Significantly higher mPAP reduction as compared to the CHAMPION trial cohorts Similar mPAP reduction in sex and LVEF subgroups Median use of the system 98.6%
Desai *et al*.^43^	Retrospective study (Medicare claims data)	2014–2015	1114	Sensor implant + monitoring	N/A	N/A	N/A	N/A	Significant reduction in HFH rate in the 6 months post‐ versus pre‐implant	HFH reduction associated with estimated reduction in HF‐related costs Similar findings in the subset of patients with complete 12‐months data available HFH reduction consistent in age, sex and type of implant subgroups
Abraham *et al*.^44^	Retrospective matched cohort study (Medicare claims data)	2014–2016	2174	Sensor implant + monitoring	No implant (matched controls)	N/A	100%	N/A	Significant reduction in total HFH at 12 months	Significant reduction in all‐cause death and composite of all‐cause death or HFH at 12 months More days alive out of the hospital Clinical benefits consistent in age, sex and comorbidities subgroups

6MWT, 6‐min walking test; CHAMPION, CardioMEMS Heart Sensor Allows Monitoring of Pressure to Improve Outcomes in NYHA Class III Heart Failure Patients; COAST, CardioMEMS HF System Post‐Market Study; COVID‐19, coronavirus disease 2019; CpcPH, combined post‐ and pre‐capillary pulmonary hypertension; CSS, clinical summary score; dPAP, diastolic pulmonary artery pressure; DSRC, device‐ or system‐related complication; GUIDE‐HF, haemodynamic‐GUIDEed management of Heart Failure; HFE, heart failure event; HFH, heart failure hospitalization; IpcPH, isolated post‐capillary pulmonary hypertension; KCCQ, Kansas City Cardiomyopathy Questionnaire; LVEF, left ventricular ejection fraction; MEMS‐HF, CardioMEMS European Monitoring Study for Heart Failure; MLHFQ, Minnesota Living with Heart Failure Questionnaire; mPAP, mean pulmonary artery pressure; N/A, not available; NT‐proBNP, N‐terminal pro‐B‐type natriuretic peptide; NYHA, New York Heart Association; OSS, overall summary score; PAP, pulmonary artery pressure; PH, pulmonary hypertension; PHQ‐9, 9‐item Patient Health Questionnaire; QoL, quality of life; RCT, randomized controlled trial; sPAP, systolic pulmonary artery pressure; TSS, total symptom score; VAS, visual analogue scale.

^a^ LVEF <40% (instead of ≤40%).

^b^ Hospitalization for decompensated HF or urgent visit with the need for intravenous diuretics.

The larger haemodynamic‐GUIDEed management of Heart Failure (GUIDE‐HF) trial enrolled 1000 patients with chronic HF, NYHA class II–IV, any LVEF and either a recent HF hospitalization within 12 months or elevated natriuretic peptides.^25^ Similarly to CHAMPION, patients were randomized to treatment arm versus control arm after successful CardioMEMS implantation. The trial had the potential to enlarge the indication to CardioMEMS to patients at lower risk than those in CHAMPION. GUIDE‐HF was, however, affected by the ongoing COVID‐19 pandemic and it was neutral with a non‐significant difference in all‐cause mortality or total HF events at 12 months (primary endpoint) between the two arms. A pre‐specified COVID‐19 sensitivity analysis showed the impact of the COVID‐19 pandemic, with a significant benefit of PAP‐guided monitoring on the primary endpoint among the patients enrolled before the COVID‐19 period, especially in those with elevated pressures at baseline during implantation.^25^, ^26^ Overall, CardioMEMS use was associated with a slightly greater (<2 mmHg) reduction in mPAP at 12 months than standard care, and no significant differences in QoL endpoints were observed. Many factors may have influenced neutral results, in addition to COVID‐19 pandemic: enrolment of a substantial proportion of relatively ‘low‐risk’ patients with baseline PAP in the target range, a reduction in mPAP through 12 months also in the control arm, and fewer patients received GDMT at 12 months as compared to baseline. Medication changes occurred more frequently in the treatment arm than in the control arm, 1.031 versus 0.608 changes per month per patient, respectively. However, changes in the cumulative doses of diuretics were not described in detail.^25^, ^27^

The recent MONITOR‐HF trial enrolled 348 patients with chronic HF, NYHA class III and a recent hospitalization or urgent visit for HF in the previous 12 months, irrespective of LVEF.^28^ They were randomized to CardioMEMS implantation with PAP monitoring or standard care without device implantation (open label). The primary endpoint of change in Kansas City Cardiomyopathy Questionnaire overall summary score (KCCQ‐OSS) from baseline to 12 months was significantly improved by CardioMEMS. The use of CardioMEMS was associated with a mean decrease of 8.4 mmHg in PAP and a significant reduction in N‐terminal pro‐B‐type natriuretic peptide levels (from 2377 to 1708 pg/ml). These changes were accompanied by significant improvements in secondary endpoints, including KCCQ‐OSS improvement or deterioration with fixed 5‐ and 10‐point thresholds, total HF hospitalizations or urgent visits (−44%), and other QoL endpoints.^28^

In addition to meta‐analyses evaluating the impact of different telemonitoring systems in HF,^17^, ^29^ a recent meta‐analysis pooled the CHAMPION, GUIDE‐HF and MONITOR‐HF trials (*n* = 1898 patients), and showed a substantial benefit of PAP‐guided haemodynamic monitoring using CardioMEMS in chronic HF with a 30% reduction in total HF hospitalizations.^30^ At subgroup analyses, there was no evidence of heterogeneity in the treatment effect across different subgroups based on age, sex, HF aetiology, LVEF phenotypes and NYHA class.^30^ In line with the three trials showing that PAP monitoring is effective across the whole range of LVEF,^22^, ^31^, ^32^, ^33^, ^34^ this meta‐analysis confirmed its efficacy in patients with either preserved or reduced LVEF, with no significant interaction between treatment arms and LVEF subgroups.^30^ Importantly, neither the three individual trials nor this meta‐analysis showed a reduction in all‐cause mortality with PAP‐guided monitoring versus standard care.^22^, ^23^, ^25^, ^28^, ^30^ Another recent patient‐level meta‐analysis pooled individual data from CHAMPION, GUIDE‐HF and Left Atrial Pressure Monitoring to Optimize Heart Failure Therapy (LAPTOP‐HF) testing a left atrial pressure (LAP) monitoring device, and demonstrated a reduction in both all‐cause mortality and HF hospitalizations among 1350 patients with HFrEF (hazard ratios [95% confidence intervals] 0.75 [0.57–0.99], *p* = 0.043, and 0.64 [0.55–0.76], *p* < 0.0001, respectively).^35^

## Observational studies

Major real‐world studies on CardioMEMS use in patients with HF are reported in *Table* *1*. The prospective CardioMEMS European Monitoring Study for Heart Failure (MEMS‐HF) enrolled 239 outpatients with NYHA class III and a HF hospitalization in the previous year, who underwent CardioMEMS implantation with PAP monitoring in Europe.^36^ Freedom from device‐ or system‐related complications and from sensor failure at 12 months were 98.3% and 99.6%, respectively. A significant reduction in annualized HF hospitalization rate was observed in the 12 months post‐ versus pre‐implantation, along with reduction in PAP and improvement in QoL endpoints.^36^ Clinical benefits were independent of presence or subtype of pulmonary hypertension.^37^

The larger prospective CardioMEMS Post‐Approval Study enrolled 1200 HF patients with similar characteristics in the United States.^38^, ^39^ Device safety, patient compliance and PAP reduction were confirmed, along with reduction in adjudicated HF hospitalizations in the 12 months after versus before implantation, sustained up to 2 years.^38^, ^39^, ^40^ Similar results were also observed in the smaller European CardioMEMS HF System Post‐Market Study (COAST) ‐ UK.^41^

Retrospective studies confirmed the reduction in mPAP and HF hospitalization rate in large US real‐world cohorts as well as small European cohorts (*Table* *1*).^42^, ^43^, ^44^, ^45^ Device‐related adverse events have been reported in the Food and Drug Administration MAUDE database, based on a mean of 41 reports per 6‐month period from May 2014 to May 2017, to a mean of 356 reports per 6‐month period after the second half of 2017. Most adverse event reports were for inaccurate measurements, which required a replacement of the external CardioMEMS unit.^46^ Although this analysis lacks granular data to evaluate the direct association between CardioMEMS and adverse events,^46^ further data from real‐world long‐term device registries seem needed.

## Indications and contraindications to invasive haemodynamic monitoring

Current 2021 ESC guidelines for the diagnosis and treatment of HF state that the CardioMEMS system may be considered to measure and monitor PAP in symptomatic patients with HF with reduced ejection fraction in order to improve clinical outcomes with a class IIb recommendation and a level of evidence B, as based on only the CHAMPION trial.^1^ However, the ESC guidelines were published before the results of GUIDE‐HF became available and, similarly, the 2023 ESC guideline focused update was also finalized before MONITOR‐HF was published.^1^, ^47^

The CardioMEMS HF system represents a significant advance in HF management, offering a tailored treatment for patients with HF. However, it is crucial to consider both medical indications and contraindications, as well as the patient's overall health profile, compliance with medical care, and the potential benefits of remote monitoring (*Table* *2*). Higher risk groups, such as NYHA class III patients and patients with a recent HF hospitalization, will most likely receive the larger absolute risk reductions. Results demonstrate consistent benefits from PAP‐guided remote patient management across the LVEF spectrum with elevated PAP despite being already on GDMT.^22^, ^25^, ^28^, ^30^ Subgroup analyses have demonstrated that the CardioMEMS system has beneficial effects across different subgroups, including patients with left ventricular assist device (LVAD), patients with cardiac resynchronization therapy (CRT), and patients with pulmonary hypertension, regardless of the subtype. Both male and female patients experienced similar reductions in HF‐related hospitalizations, which is reassuring given known disparities in HF treatment by sex.^37^, ^48^, ^49^, ^50^ The efficacy was also independent of LVEF.

**Table 2:** Indications and contraindications to invasive haemodynamic monitoring with the CardioMEMS system

**Indications**
General conditions
NYHA class II or III
One worsening HF event (hospitalization or unplanned visit) in the previous 12 months or elevated high natriuretic peptide plasma concentrations^a^
Additional conditions
Patients on GDMT
Difficulty in managing or assessing fluid volumes
Challenging physical assessment (e.g. COPD)
Compliance with HF medical care
Benefiting from remote monitoring (especially those living far from clinics)
**Contraindications**
Inability to take dual antiplatelet or anticoagulant therapy for 1 month post‐implant
Active infection
History of recurrent PE or DVT
Inability to tolerate RHC
GFR <25 ml/min, non‐responsive to diuretics, or on chronic renal dialysis
Congenital heart disease or mechanical right heart valve(s)
Coagulation disorders
Hypersensitivity or allergy to ASA and/or clopidogrel
Recent (within 3 months) implantation of a CRT
Major cardiovascular event, including pulmonary embolism, in the last 2 months
BMI >35 kg/m^2^, chest circumference >165 cm at axillary level

ASA, aspirin; BMI, body mass index; BNP, B‐type natriuretic peptide; COPD, chronic obstructive pulmonary disease; CRT, cardiac resynchronization therapy; DVT, deep vein thrombosis; GDMT, guideline‐directed medical therapy; GFR, glomerular filtration rate; HF, heart failure; LVEF, left ventricular ejection fraction; NT‐proBNP, N‐terminal pro‐B‐type natriuretic peptide; NYHA, New York Heart Association; PE, pulmonary embolism; RHC, right heart catheterization.

^a^ BNP ≥250 pg/ml or NT‐proBNP ≥1000 pg/ml with threshold corrections for LVEF and BMI (4% reduction per BMI unit over 25 kg/m^2^):
LVEF ≤40%: NT‐proBNP ≥1000 pg/ml (or BNP ≥250 pg/ml) LVEF >40%: NT‐proBNP ≥700 pg/ml (or BNP ≥175 pg/ml).

Contraindications include patients who cannot take antiplatelet or anticoagulant therapy post‐implantation and those with conditions like active infections, history of recurrent pulmonary embolism or deep vein thrombosis. Additionally, the treatment is not advised for patients with severe kidney dysfunction, congenital heart disease, coagulation disorders, hypersensitivity to certain medications, recent CRT implantation, or those who present certain physical parameters such as a high body mass index and chest circumference.^51^

**Figure 1:**
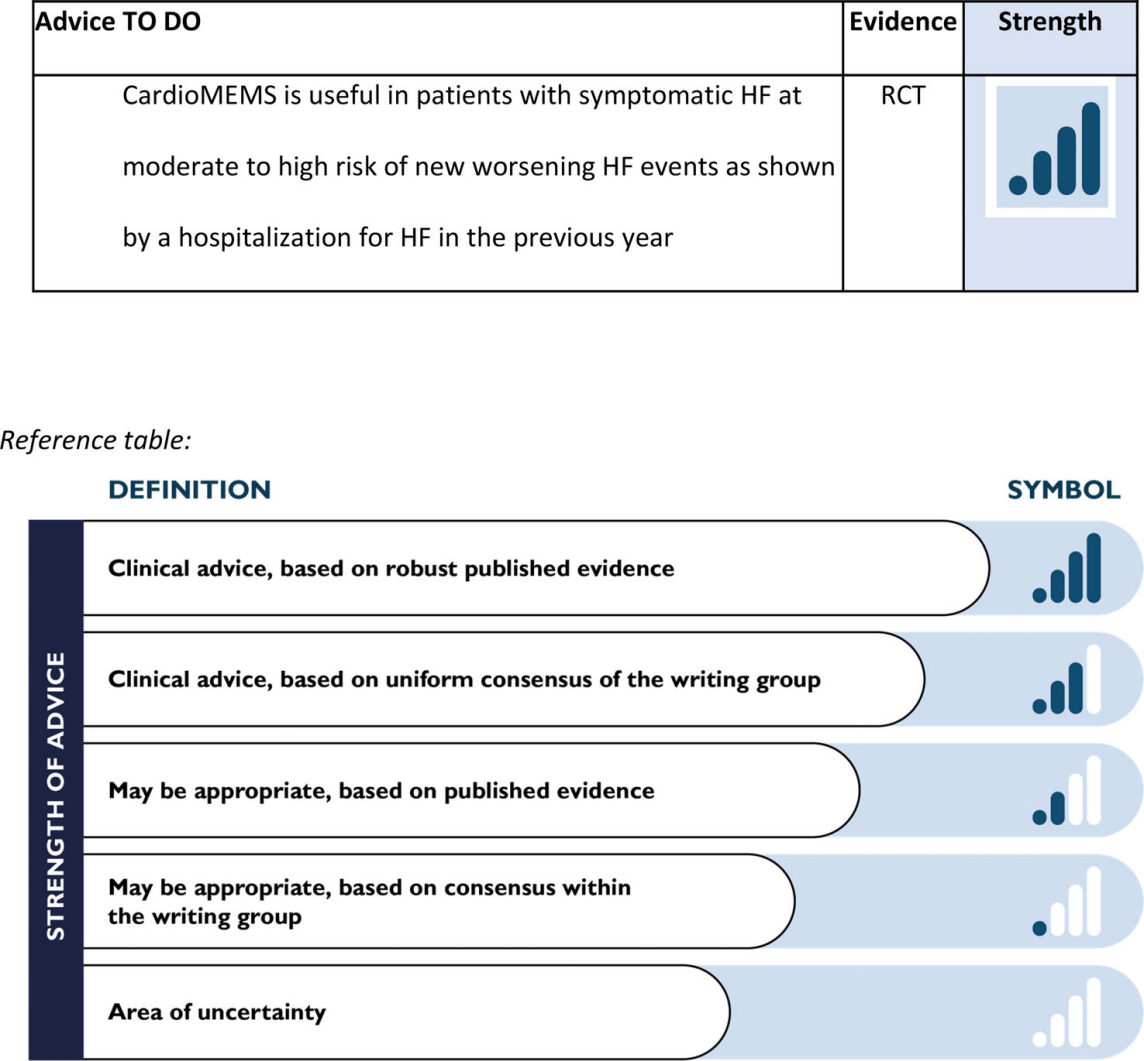
Table of advice. HF, heart failure; RCT, randomized controlled trial.

**Figure 2:**
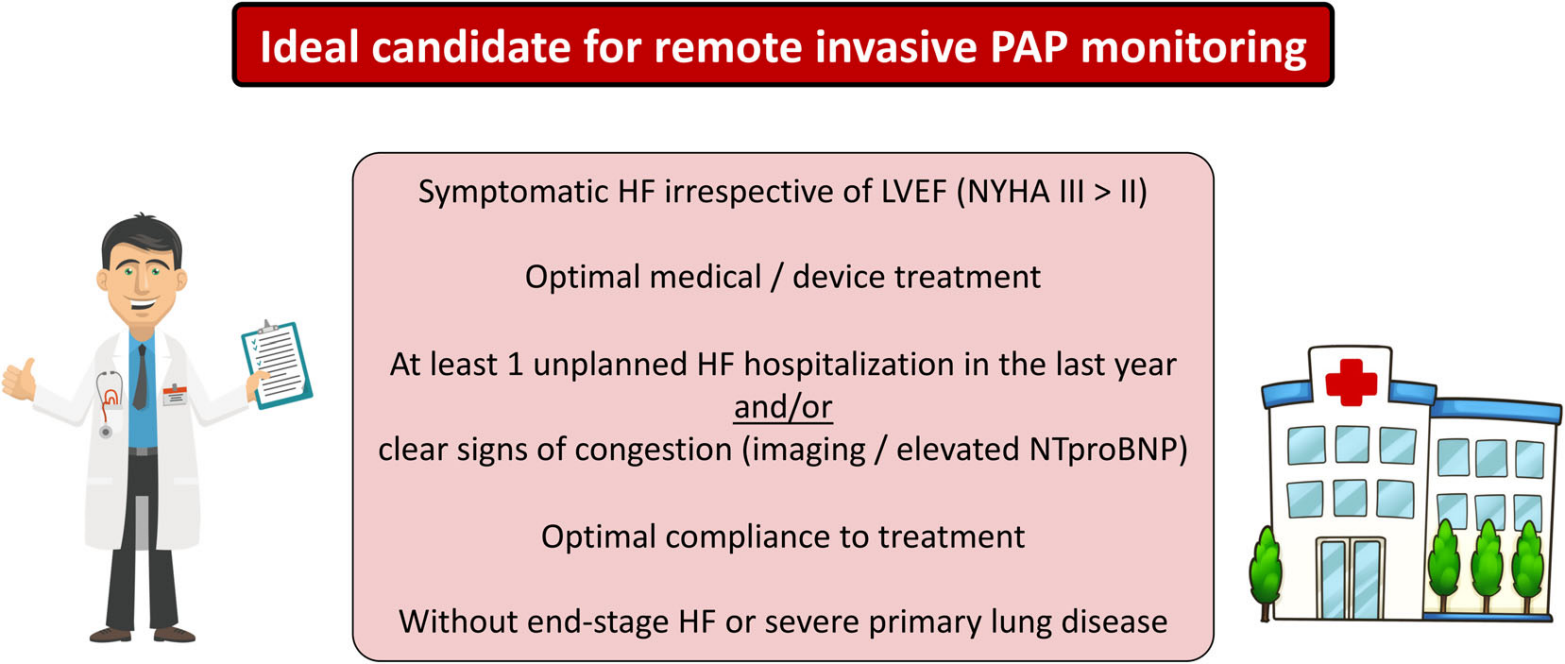
Ideal candidate for remote invasive pulmonary artery pressure monitoring. HF, heart failure; LVEF, left ventricular ejection fraction; NYHA, New York Heart Association; NTproBNP, N‐terminal pro‐B‐type natriuretic peptide; PAP, pulmonary artery pressure. *Further evidence needed in NYHA class II patients. **Contraindications to CardioMEMS implantation include intolerance to antiplatelet or anticoagulant therapy post‐implantation, active infections, history of recurrent pulmonary embolism or deep vein thrombosis.

Based on the available data, the CardioMEMS system is useful for patients with symptomatic HF at moderate to high risk of new worsening HF events as shown by one or more worsening HF events in the previous year (*Figure* *1*). The characteristics of the ideal candidate for remote invasive PAP monitoring are depicted in *Figure* *2*.

## Practical management

Before CardioMEMS implantation, patients with HF should receive optimal or maximum tolerated GDMT according to ESC guidelines and be evaluated for an implantable cardioverter‐defibrillator (ICD) or CRT if indicated.^1^ Of note, practical management strategies before, during and after CardioMEMS implantation are derived from available clinical trials, but no randomized trials comparing different PAP monitoring practices have been performed so far.

**Figure 3:**
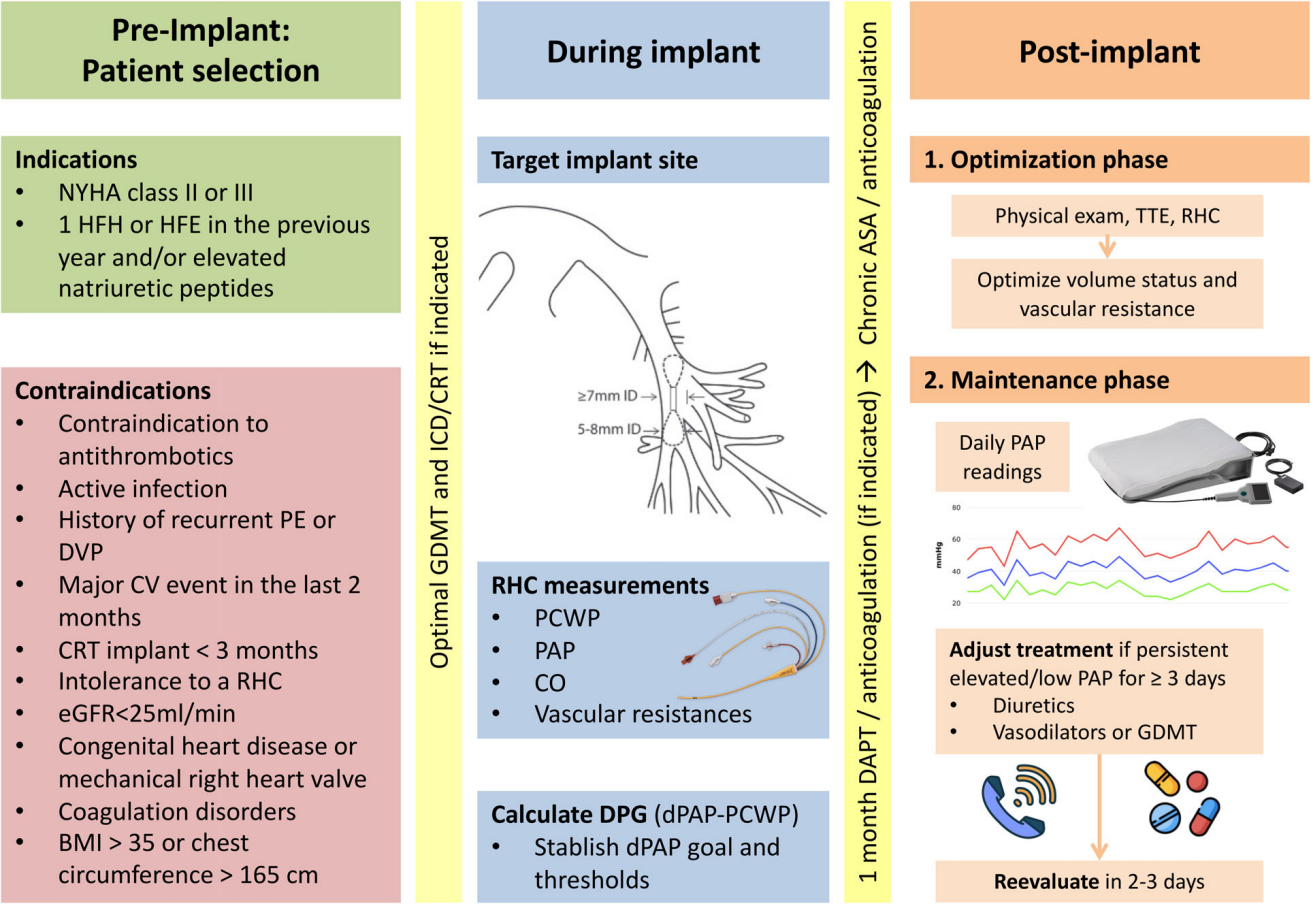
‘What‐to‐do’ steps for implementation of CardioMEMS. ASA, aspirin; BMI, body mass index; BNP, B‐type natriuretic peptide; CO, cardiac output; CRT, cardiac resynchronization therapy; CV, cardiovascular; DAPT, dual antiplatelet therapy; dPAP, diastolic pulmonary artery pressure; DPG, diastolic pulmonary gradient; DVT, deep vein thrombosis; eGFR, estimated glomerular filtration rate; GDMT, guideline‐directed medical therapy; HF, heart failure; HFE, heart failure event; HFH, heart failure hospitalization; ICD, implantable cardioverter‐defibrillator; LVEF, left ventricular ejection fraction; NYHA, New York Heart Association; NT‐proBNP, N‐terminal pro‐B‐type natriuretic peptide; PAP, pulmonary artery pressure; PCWP, pulmonary capillary wedge pressure; PE, pulmonary embolism; RHC, right heart catheterization.

A right heart catheterization is performed during device implantation (*Figure* *3*). It is important to accurately measure pulmonary capillary wedge pressure (PCWP), PAP, cardiac output, and vascular resistances at baseline as they will be the reference for further changes in treatment and are used to calibrate the sensor. The diastolic PAP is crucial, as it is the primary management parameter. The difference between diastolic PAP and PCWP must be carefully noted, and a diastolic pressure gradient ≥5 mmHg may indicate pre‐capillary pulmonary hypertension, which is essential for establishing PAP thresholds to avoid over‐diuresis.

Pre‐implant the patient or caregiver must be trained to take daily readings, emphasizing the clinical importance of these measurements, since patients who will be non‐compliant to the measurement will not benefit from pressure‐guided management. Patients with elevated intravascular volume and increased PAP benefit from increased doses of loop diuretics, combined diuretic therapy, optimization of GDMT and direct vasodilators. Of note, although neurohormonal antagonists are usually titrated based on well‐known titration schemes and target doses are derived from pivotal randomized trials and recommended in guidelines, there are no specific recommendations for titration of diuretics and direct vasodilators in HF, and PAP guidance offers a useful way to tailor these medications. In this regard, although changes in diuretic doses are the most common drug intervention in response to changes in PAP, the addition or titration of direct vasodilators was also part of the treatment algorithm used in randomized trials on PAP‐guided management.^22^, ^23^, ^25^, ^28^ However, further studies specifically investigating the optimal algorithms for treatment optimization in response to PAP monitoring are awaited.

After optimizing medical treatment, monitoring should be aimed at maintenance of target diastolic PAP (*Table* *3*). Achieving and maintaining long‐term stability by keeping PAP in the target range on a day‐to‐day basis is the crucial intervention to improve patients' QoL and reduce the risk of HF‐related hospitalizations. Treatment aims to maintain diastolic PAP within normal values, with a usual upper target of 16–20 mmHg and an ideal target range between 8 and 20 mmHg, by adjusting medications, mainly diuretics and GDMT and also vasodilators in some cases.^52^, ^53^ Conversely, persistently low PAP might lead to a dose reduction or discontinuation of diuretic therapy with further reassessment to ensure no rise occurs in the following days. Likely, a part of the success of PAP‐guided optimization of GDMT lies within timely down‐titration of diuretics and other drugs, preventing forward failure and renal dysfunction. The personalized up‐titration or down‐titration of diuretics and/or vasodilators offers additional room for careful titration of HF‐modifying drugs, specifically renin–angiotensin–aldosterone system inhibitors, beta‐blockers, and mineralocorticoid receptor antagonists, allowing even further optimal titration in vulnerable HF patients.

**Table 3:** How to monitor and treat patients during follow‐up after pulmonary artery pressure monitoring device implantation

Telemonitoring with daily check (5/7 days) by HF personnel^a^
Carefully establish baseline values corresponding to absence of congestion and clinical stability in the first weeks after implantation
Monitor trends over 3–5 days
Have telephone calls when meaningful increase in trend
Treat targeting the cause of increase Pressure without volume increase Titrate neurohormonal modulators, vasodilators, SGLT2 inhibitors Pressure with volume increase Titrate neurohormonal modulators, vasodilators, SGLT2 inhibitors Titrate loop diuretics (×2 for 3 days) Adopt combined diuretic therapy (metolazone, thiazides, acetazolamide, etc.) Persistent pressure increase Consider additional therapies when indicated (valve interventions, PV isolation for AF, LVAD, etc.)

AF, atrial fibrillation; HF, heart failure; LVAD, left ventricular assist device; PV, pulmonary vein; SGLT2, sodium–glucose cotransporter 2.

^a^ Not all European countries have nurses available.

Since PAP typically rises several days before patients exhibit overt congestion,^4^, ^5^, ^6^ it is advised to intervene once the patient is noted to have a 3–5 mmHg change in diastolic PAP over 2–3 days or a change of 5 mmHg or more in a single day, with a re‐evaluation in 2–3 days. It is important to look for the precipitating factors that might have contributed to a change in PAP. If PAP remains high, other causes such as dietary transgression, sleep apnoea, anaemia, arrhythmias (atrial fibrillation), new valvular heart disease, or HF progression should be investigated. Finally, it is important to understand that the relationship between PAP and volume status may be different in each patient and volume assessment via other means may be advised in some patients.^54^, ^55^

## Specific settings

### Patients with cardiac implantable electronic devices

Pacemakers, ICDs, and LVADs are designed to be compatible with the pulmonary artery sensor used in the CardioMEMS system and do not hinder its performance. Additionally, patients who have received a CRT can also be considered for the CardioMEMS system, provided at least 3 months have passed since the CRT implantation.^51^

The simultaneous use of the CardioMEMS system with ICD and CRT devices can significantly enhance patient management in HF. Continuous monitoring by CardioMEMS may provide early indications of haemodynamic changes that require adjustments in therapy or device settings, enhancing patient outcomes with more personalized device adjustments. Additionally, CardioMEMS can aid arrhythmia management by offering early signals that might precede arrhythmias. On the other hand, CRTs and ICDs may give data on current heart rhythm showing, for instance, new‐onset atrial fibrillation that may cause haemodynamic worsening.

By integrating the active management features of CardioMEMS with the therapeutic actions of ICDs and CRTs, hospitalizations due to HF or related complications may be reduced, thus establishing a more integrated and effective approach to care.

### Patients with cardiac transplantation and left ventricular assist device

Despite maximal GDMT and haemodynamic‐guided care, clinical disease progression occurs in HF patients, in many cases requiring LVADs or heart transplant. LVAD has been an increasingly utilized therapy for advanced HF and resulted in improvements in patient functional status and QoL, in addition to improved clinical outcomes relative to medical therapy.^56^, ^57^ There is a potentially important clinical role for remote haemodynamic monitoring in clinical decision‐making and patient management before and after LVAD implantation.^58^

A sub‐analysis of the CHAMPION trial suggested utility in using these devices to improve the timing of LVAD implantation, although further information is needed in this area.^48^ These patients who are not yet sick enough to warrant an LVAD but have an elevated PAP may require more frequent monitoring and medical adjustments in order to prevent decompensation and/or consider earlier LVAD implantation.

Using haemodynamic information leads to better optimization and better decongestion and unloading of the right ventricle, potentially reducing the risk of right ventricular failure after LVAD implantation. Also, changes in diastolic PAP after LVAD implantation might indicate potential post‐operative complications.^59^ The ability to remotely and non‐invasively monitor PAP is attractive in a population of HF patients who are not only on anticoagulation, but also supported by mechanical circulatory support with a propensity for bleeding. Moreover, early detection of changes in PAP and heart rate by CardioMEMS can alert clinicians to the pre‐clinical phase of complications like gastrointestinal bleeding, common in LVAD patients.^60^ Another advantage of remote monitoring is the ability to distinguish a subset of patients who may have reversible pulmonary hypertension from those who have a fixed component that does not respond to pharmacological or mechanical interventions. This may be especially valuable for patients receiving an LVAD as a bridge to transplant eligibility in the presence of pulmonary hypertension.^61^

In the largest, prospective observational study of patients with continuous‐flow LVAD and implantable haemodynamic PAP monitoring devices, the CardioMEMS system was able to provide a longitudinal haemodynamic profile with a high degree of patient compliance. Relatively modest reductions in diastolic PAP (3–5 mmHg) were associated with significant improvements in functional capacity. Furthermore, among patients whose diastolic PAP was maintained less than 20 mmHg, fewer HF hospitalizations were observed.^62^ Additional studies have demonstrated that haemodynamic optimization using ramp protocols is associated with reduced hospitalizations and improved functional capacity in LVAD patients.^63^, ^64^, ^65^, ^66^, ^67^ However, ramp studies reflect conditions at a single time point. By contrast, the CardioMEMS system can provide a longitudinal haemodynamic profile of patients supported with LVADs.

## Heart failure team organization and telemonitoring pathways

The management of HF has significantly evolved with the advent of telemonitoring technologies like CardioMEMS. This system requires a reorganization of the HF care units to effectively integrate PAP monitoring in the team's protocol, which represents a paradigm shift with a more proactive approach. The team should include cardiologists and dedicated nurses or technicians trained in interpreting pulmonary artery data. These professionals must collaborate to ensure continuous monitoring and timely interventions before clinical deterioration occurs. The provision of such staff is a requirement for the implementation of PAP monitoring systems, such as CardioMEMS. This process requires a precise identification of the specific tasks of all staff members, guidelines on their training (which need to be incorporated into the respective country's education and training scheme), and a clear definition of the job positions along with the corresponding reimbursements.

Dedicated nurses play a pivotal role, regularly reviewing data from the CardioMEMS system and communicating with patients to adjust medications or lifestyle recommendations as needed, with the support of the cardiologist for challenging cases. There is a need to check PAP several days a week but PAP usually rise days to 2 weeks before HF decompensation occurs. A notification tool showing only patients out of optimal range and with missing readings is available within the CardioMEMS system, and can be used to optimize the time dedicated to data reviewing. This constant feedback loop ensures that patients receive personalized care, tailored to their specific needs and changes in their condition. Additionally, this model promotes patient engagement and self‐management, essential components of successful chronic disease management.

The cost‐effectiveness of CardioMEMS is another critical consideration. Previous studies have shown that while the initial investment in the technology may be significant, the long‐term savings from reduced hospitalizations and emergency visits are substantial.^68^, ^69^, ^70^, ^71^ Moreover, improved QoL and patient satisfaction are benefits that complement the financial advantages.^28^ Of note, reimbursement policies for device implantation and subsequent follow‐up vary in different countries, and this might have relevant implications for the adoption of this technology in the real world.

For the successful implementation of CardioMEMS, a clear ‘what‐to‐do’ guide is essential. This includes a pre‐procedural assessment to evaluate patient suitability, intra‐ and peri‐procedural management to ensure safe and effective implantation, and comprehensive post‐procedural care focusing on patient education and regular monitoring. Such a guide would serve as a valuable resource for HF teams, ensuring standardized, high‐quality care for patients undergoing CardioMEMS implantation (*Figure* *2*). Future dedicated studies are needed to further refine the pre‐, peri‐ and post‐procedural management and optimize the treatment algorithms in these patients.

## New devices for invasive haemodynamic monitoring

In the first‐in‐human SIRONA study, the commercially‐available Cordella Heart Failure System was combined to the novel investigational Cordella PAP Sensor (Endotronix Inc, Chicago, IL, USA) to remotely monitor vital signs and PAP, and the feasibility of this comprehensive telemonitoring strategy was evaluated in 15 patients with HF and NYHA class III.^72^ The sensor combined with the Cordella Heart Failure System (remote monitoring of weight, heart rate, oxygen saturation, and blood pressure) allows a more comprehensive patients' telemonitoring. Sensor implantation was feasible and safe, and the device accurately measured PAP in combination with daily transmission of vital signs.^72^ Subsequently, the SIRONA 2 trial confirmed device safety and efficacy in measuring mPAP among 70 HF patients.^73^ Recently, the results of the single‐arm, open‐label PROACTIVE‐HF study enrolling 456 HF patients with NYHA class III have been reported, showing that remote monitoring of seated PAP with the Cordella sensor, combined with assessment of vital signs with the Cordella Heart Failure System, was safe, reduced 6‐month incidence of death or HF hospitalization compared to a performance goal, improved QoL and functional capacity, and enabled significant PAP reduction among patients with elevated PAP at baseline.^74^, ^75^ These findings were hypothesis‐generating and the ongoing PROACTIVE‐HF‐2 randomized trial will further assess the safety and efficacy of the Cordella system, along with a dedicated evaluation of a clinician‐directed patient self‐management strategy (NCT05934487).

The first‐in‐human V‐LAP Left Atrium Monitoring systEm for Patients With Chronic sysTOlic & Diastolic Congestive heart Failure (VECTOR‐HF) study demonstrated the safety, feasibility and technical performance of the novel V‐LAP system (Vectorious Medical Technologies, Tel‐Aviv, Israel).^76^, ^77^ V‐LAP is a leadless implantable sensor that is positioned in the interatrial septum and is able to measure and remotely transmit LAP. Device implantation was safe and sensor‐calculated LAP had a good correlation with invasive PCWP.^76^, ^77^ The ongoing VECTOR‐HF II study will further test this technology coupled with a dedicated patient self‐management app.^77^

Other haemodynamic monitoring devices have been previously tested in HF. The Chronicle implantable haemodynamic monitor consists of a transvenous single‐lead connected to a programmable device, that is able to continuously measure and store right ventricular pressure and estimated diastolic PAP, and that was tested in HF either alone or combined with an ICD.^20^, ^78^, ^79^ The HeartPOD device (St. Jude Medical, Minneapolis, MN, USA) is a LAP monitoring system consisting of a transvenous transseptal left atrial lead connected to a subcutaneous antenna coil.^80^ Despite a reduction in HF hospitalizations with HeartPOD, the LAPTOP‐HF trial was prematurely stopped due to an excess of procedure‐related complications.^81^

## Gaps in knowledge and future perspectives

Although it has been tested in randomized trials,^22^, ^23^, ^25^, ^28^ further real‐world assessment of CardioMEMS safety and performance, especially in the long‐term follow‐up, is warranted. Furthermore, the identification of the clinical profile of ‘responders’ who may benefit the most from PAP monitoring could avoid futile implantation procedures, enhance cost‐effectiveness and optimize outcomes. Some randomized trials are ongoing to test CardioMEMS in specific settings, such as NYHA class III HF (NCT04398654), advanced HF (NCT05284955), cardiogenic shock survivors (NCT04419480), or HF management in the context of a virtual HF clinic (NCT04441203). Beyond CardioMEMS, rigorous clinical trials testing and validating other haemodynamic monitoring devices, including Cordella and V‐LAP, are needed.

Pressure‐based monitoring systems can detect volume overload above the stressed blood volume threshold, whereas other strategies may be more sensitive in capturing modest increases in unstressed blood volume, that is before causing any increase in circulatory filling pressure.^82^ Early pre‐clinical studies evaluated the safety and performance of the novel implantable FIRE1 System (Foundry Innovation & Research 1 Ltd., Dublin, Ireland), that allows real‐time remote monitoring of inferior vein cava (IVC) area and may therefore be particularly useful in HF.^83^, ^84^ Interestingly, changes in IVC area were more sensitive than changes in filling pressures.^83^, ^84^ However, this strategy needs to be properly tested in the clinical setting.

The development of novel technologies may also expand the armamentarium of remote haemodynamic monitoring devices. However, beyond the performance of the individual device, a multiparametric approach combining different systems might be particularly useful to refine the home‐based HF management.^85^ Importantly, the clinical performance of these new technologies, such as PAP‐guided or LAP‐guided management and IVC measurement, should be closely monitored in dedicated nationwide or European monitoring registries.

## Conclusions

Randomized trials and observational studies have demonstrated that remote PAP‐guided management with CardioMEMS reduces HF‐related hospitalizations in patients with chronic HF. Implantation of this device may be appropriate in patients with HF (NYHA class II or III), who have had one or more HF event in the previous year and/or elevated natriuretic peptides. Standardization of the procedure, of pre‐, peri‐ and post‐procedural care, and of a pharmacological algorithm are key aspects to deliver remote PAP‐guided management safely and effectively in the real world. Additional studies are needed to further explore the usefulness of this strategy, refine its management, and test novel devices for haemodynamic monitoring.
